# Emergency cancer diagnosis in Paris: A cross‐sectional study using AP‐HP data

**DOI:** 10.1002/ijc.70056

**Published:** 2025-08-08

**Authors:** Matthew E. Barclay, Ariel Cohen, Sonia Priou, Marie Verdoux, Rémi Flicoteaux, Alexis Laurent, Gilles Chatellier, Christophe Tournigand, Georgios Lyratzopoulos, Emmanuelle Kempf, Guillaume Lamé

**Affiliations:** ^1^ Epidemiology of Cancer Healthcare and Outcomes, Research Department of Behavioural Science and Health Institute of Epidemiology and Health Care, University College London London UK; ^2^ Innovation and Data, IT Department Assistance Publique—Hôpitaux de Paris Paris France; ^3^ Laboratoire Génie Industriel, CentraleSupélec Université Paris‐Saclay Gif‐sur‐Yvette France; ^4^ Department of Medical Information Assistance Publique—Hôpitaux de Paris Paris France; ^5^ Department of Digestive Surgery Henri Mondor and Albert Chenevier University Hospital, Assistance Publique—Hôpitaux de Paris, Université Paris Est Créteil Créteil France; ^6^ INSERM U955, Equipe 18 Institut Mondor de Recherche Biomédicale Créteil France; ^7^ Department of Medical Informatics Assistance Publique Hôpitaux de Paris, Centre‐Université de Paris (APHP‐CUP), Université Paris CIté Paris France; ^8^ Department of Medical Oncology Henri Mondor and Albert Chenevier University Hospital, Assistance Publique—Hôpitaux de Paris, Université Paris Est Créteil Créteil France; ^9^ Laboratoire d'Informatique Médicale et d'Ingénierie des Connaissances pour la e‐Santé (LIMICS) Sorbonne University Inserm, Université Sorbonne Paris Nord Paris France

**Keywords:** diagnosis, electronic health records, emergency, Paris

## Abstract

Many cancer patients are diagnosed following an emergency hospital admission in the 30 days preceding their diagnosis (‘emergency presentation’) and these patients have worse outcomes. Despite multiple international studies, there is only sparse evidence on emergency diagnosis of cancer in France. We examined the frequency of emergency presentation and its association with survival in patients with breast, lung, colon, rectal and pancreatic cancer, using data from the Paris Region University Hospitals (Assistance Publique Hôpitaux de Paris, AP‐HP). Between January 2019 and June 2022, 25,845 patients were newly referred with a relevant cancer, of whom 3960 (15.3%) were emergency presentations (23% for pancreatic, 20% for colon, 19% for lung, 9% for rectal and 6% for breast cancer). Emergency presenters were more likely to have metastatic cancer, less likely to have surgical treatment (except for colon cancer patients), and more likely to die within a year from diagnosis. The risk of emergency presentation increased with age and was higher for lung and pancreatic cancer. Emergency presentation rates for lung cancer were highest in the first Covid‐19 pandemic year (2020). Emergency presentation was strongly associated with worse survival compared with non‐emergency presentation (HR 2.67, 95% CI 2.52–2.84), even after adjustment for metastatic status. Emergency presentation is a common route to diagnosis in AP‐HP and can be identified from routine datasets. Efforts to reduce diagnosis as an emergency may help improve cancer outcomes, justifying public health and health system initiatives supporting prompt presentation and diagnosis.

## What's New?

Research in a small number of high‐income countries suggests that cancer diagnosis resulting from an emergency department visit is associated with poor clinical outcomes. Here, this relationship was examined specifically in France, where little is known about emergency cancer diagnosis. Analyses of data from Paris Region University Hospitals show that some 15% of cancers diagnosed between 2019 and 2022 were associated with emergency presentations. Emergency patients were at increased risk of presenting with metastatic cancer and of dying within 1 year of diagnosis. The findings support the conclusion that emergency presentation of cancer is strongly associated with dismal survival.

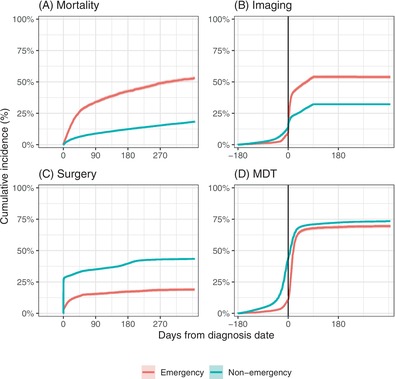

**Abbreviations**
AP‐HP: Assistance Publique Hôpitaux de Paris
CI: confidence interval
CT: computed tomography
EP: ‘emergency presentation’, defined in this study as diagnosis within 30 days of an emergency hospital admission
HR: hazard ratio
MDT: multi‐disciplinary team
NLP: natural language processing
OR: odds ratio

## 1 INTRODUCTION

A recent international study including six high income countries has shown that diagnosis of cancer as an emergency is common and associated with poor outcomes.^1^ The proportion of cancers that are ‘Emergency Presentations’ (EPs) varies between countries and cancer sites, but this diagnostic route is consistently associated with certain patient or tumour characteristics (such as older age, and advanced stage disease), and poorer outcomes compared with patients diagnosed through elective care routes. Several single‐country studies provide similar findings.^2^, ^3^, ^4^ The frequency of emergency presentations has been examined in France only in relatively small studies of lung and colorectal cancer,^5^, ^6^ despite research examining emergency department attendance by cancer patients post‐diagnosis.^7^, ^8^, ^9^ We aimed to enhance this evidence base by considering five common cancers, larger sample sizes, and recent diagnosis periods, and acquire insights about the nature of emergency diagnosis of cancer in the context of a universal health system with certain unique features.

This study had three linked aims. First, to apply a definition of emergency presentation that can be estimated from French hospital claims data, and examine associations with peri‐diagnostic activity such as in‐hospital imaging, multi‐disciplinary team discussion, and surgery with curative intent. We have chosen Paris hospitals as the study setting given the availability of high‐quality electronic patient records for hospitalised patients. Related to this aim, we have assessed the construct validity of our definition regarding associations with patient and tumour characteristics expected from prior, other country, evidence. Second, to estimate the proportion of newly referred patients with cancer to a large hospital network in Paris who presented as emergencies, and identify patient groups at higher risk of such presentation. Third, to describe associations between emergency presentation and cancer survival up to 1 year.

## 2 METHODS

### 2.1 Dataset

Assistance Publique Hôpitaux de Paris (AP‐HP) is the university hospital trust for Paris and the surrounding areas; it includes 38 constituent hospitals. AP‐HP is not the only cancer care provider in the Paris region; public non‐university hospitals, private hospitals, and specialised cancer care centres (Institut Curie and Gustave Roussy) also provide cancer care in the area. AP‐HP treats around one third of new cancer cases in the Paris region (around 60,000 patients each year). We analysed data from AP‐HP's Clinical Data Warehouse,^10^ which contains information on more than 11 million patients treated at AP‐HP since 2017. Among the available information, we used electronic health records (imaging reports, multi‐disciplinary team meeting reports) and claims data.

We considered only patients with a single cancer diagnosis in the database and study period 2019–2022 of one of five cancer sites: breast, lung, colon, rectum, and pancreas (codelists in Table S1). We defined ‘cancer diagnosis’ as the first record of cancer in the AP‐HP coded claims data, with at least 2 years of data beforehand without a record of any cancer at AP‐HP. Information on cancer‐related care received outside of AP‐HP was not available in this project, thus patients who were not treated at AP‐HP hospitals were not included.

### 2.2 Outcomes

Our primary outcome was ‘emergency presentation’, defined as a newly referred cancer diagnosis with an emergency admission to hospital in the preceding 30 days.

Additionally, to estimate the survival of cancer patients, from diagnosis to 365 days after diagnosis, we identified vital status from hospital records and from linkage to national mortality data. The last available update to national mortality data was to June 2022, and data was, therefore, censored on 1 June 2022 if patients were not known to have died according to the hospital records.

Finally, we explored some measures of care activity. First, curative surgical treatment, between diagnosis and 365 days after diagnosis, identified from claims data using appropriate codelists (Table S2). Second, discussion at a Multi‐Disciplinary Team (MDT) meeting, between 180 days before diagnosis and 365 days after diagnosis, identified from text documents in the electronic health record using a natural language processing (NLP) algorithm.^11^ Third, in‐hospital imaging (CT‐scans only, this being the standard imaging procedure to assess cancer spread at diagnosis for the sites included in this study), between 180 days before diagnosis and 90 days after diagnosis, similarly identified using an NLP algorithm from documents in the electronic health record.^11^ In both cases (MDT reports and imaging), we used rule‐based algorithms that searched for keywords in free‐text reports to identify CT‐scan reports from all imaging reports and MDT reports from uncategorised documents in the patient record.

### 2.3 Covariates

For assessing prevalence and association with survival, we considered the following covariates:

- Age at diagnosis (years, linear spline with knots at 60, 70, 80; knot locations chosen to allow for non‐linear age impact in age ranges where cancer diagnosis is common).
- Sex (binary, male/female).
- Diagnosis year (2019, 2020, 2021, 2022), intended to capture possible changes due to the Covid‐19 pandemic.
- Cancer site (breast, lung, colon, rectum, pancreas).
- Metastatic status (in the survival analysis only), identified from CT‐scan reports using an NLP algorithm previously validated for all sites (also a rule‐based algorithm that looks for keywords in the free‐text reports, accounting for negation).^11^, ^12^, ^13^, ^14^

To elucidate possible associations between hospital volume and emergency presentation case‐mix, we further explored associations between the numbers of cancers diagnosed at a hospital and the proportion of those cancers that were emergency presentations using scatter plots.

### 2.4 Statistical analyses

Initial analyses described the proportion of patients who were emergency presentations, both overall and within individual cancer sites. Logistic regression was then used to calculate adjusted odds of emergency presentation for the five sites combined (adjusting for cancer site) and within each cancer site.

Associations with outcomes were described using cumulative incidence curves. For overall survival, we used the Kaplan–Meier estimator. For surgery, MDT discussion, and imaging events, we used the Aalen‐Johansen estimator, treating death as a competing risk.^15^ Cox proportional‐hazards regression models were used to estimate adjusted associations with survival.

We performed five different analyses of survival, considering metastatic status in different ways. The first analysis ignored metastatic status. The second only included patients with known metastatic status based on imaging reports. The third included all patients, considering metastatic cancer if it was mentioned on imaging reports or if they had a metastatic cancer code in their hospital claims data. For the fourth and fifth models, we accounted for missing metastatic status information using multiple imputation. For the fourth model, we created five imputed datasets based on the death indicator, the Nelson‐Aalen estimator of cumulative hazard,^16^ and the other variables in the survival model (age, sex, cancer site). For the fifth model, we additionally included records of metastatic status from the hospital claims data as auxiliary information.

To assess the representativeness of our sample, we compared cancer incidence rates in our population with Globocan estimates of cancer incidence by age for France. We calculated crude, age‐specific, and age‐standardised (to Globocan‐France) estimates of rates of emergency presentation by site and compared them to Globocan data.

Data extraction was carried out in Python, using py.spark to access the SQL database and Pandas for data processing. Data analysis was carried out in R, using the packages tidyverse,^17^ gtsummary,^18^ ggsurvfit,^19^ mice^20^ and patchwork.^21^

## 3 RESULTS

The analysis cohort included 25,845 patients with cancer (30% lung, 29% breast, 20% colon, 13% pancreas, 8% rectum; Table 1), of whom 3960 (15%) were diagnosed after an emergency presentation.

**TABLE 1:** Characteristics of breast, colon, rectum, pancreas and lung cancer patients diagnosed at AP‐HP between January 2019 and June 2022.

		All patients		Non‐emergency		Diagnosed after emergency presentation	
		*N*	(col %)	*N*	(row %)	*N*	(row %)
Total		25,845		21,885	(84.7%)	3960	(15.3%)
Cancer site	Breast	7492	(29.0%)	7055	(94.2%)	437	(5.8%)
	Colon	5147	(19.9%)	4104	(79.7%)	1043	(20.3%)
	Rectum	1999	(7.7%)	1817	(90.9%)	182	(9.1%)
	Pancreas	3483	(13.5%)	2677	(76.9%)	806	(23.1%)
	Lung	7724	(29.9%)	6232	(80.7%)	1492	(19.3%)
Sex	Female	15,477	(59.9%)	13,552	(87.6%)	1925	(12.4%)
	Male	10,368	(40.1%)	8333	(80.4%)	2035	(19.6%)
Age	18–64	11,676	(45.2%)	10,372	(88.8%)	1304	(11.2%)
	65–74	7461	(28.9%)	6411	(85.9%)	1050	(14.1%)
	75–84	4692	(18.2%)	3814	(81.3%)	878	(18.7%)
	85–99	2016	(7.8%)	1288	(63.9%)	728	(36.1%)
				***N***	**(col %)**	***N***	**(col %)**
Metastatic status (imaging reports)	Not metastatic	4543	(51.2%)	3690	(54.5%)	853	(40.6%)
	Metastatic	4330	(48.8%)	3083	(45.5%)	1247	(59.4%)
	Unknown	16,972		15,112		1860	
Surgery within 1 year	No	16,228	(62.8%)	12,984	(59.3%)	3244	(81.9%)
	Yes	9617	(37.2%)	8901	(40.7%)	716	(18.1%)
Discussed at multidisciplinary team meeting	No	7355	(28.5%)	6116	(27.9%)	1239	(31.3%)
	Yes	18,490	(71.5%)	15,769	(72.1%)	2721	(68.7%)
Died within 1 year	No	20,469	(79.2%)	18,451	(84.3%)	2018	(51.0%)
	Yes	5376	(20.8%)	3434	(15.7%)	1942	(49.0%)

The proportion of emergency presenters (EP) varied by cancer site, being 6% for breast cancer (437/7492), 9% for rectal cancer (182/1999), 19% for lung cancer (1492/7724), 20% for colon cancer (1043/5147), and 23% for pancreatic cancer (806/3483).

The cohort included 11,676 (45%) patients aged 18–64 at diagnosis; EP rates varied by age, being 11% for those aged 18–64 and 36% for those aged 85–99. A third of patients had known metastatic status at diagnosis from imaging reports, with EP patients more likely to have known metastatic status (53% vs. 31%) and more likely to have metastatic cancer if known (among patients with available metastatic status, 59% of those diagnosed after an emergency presentation had metastatic cancer vs. 46% of those diagnosed by other routes). See Tables S3–S7 for descriptive statistics by cancer site.

Median follow‐up, censored at 365 days, was 365 days (IQR 113–365 days).

**FIGURE 1:**
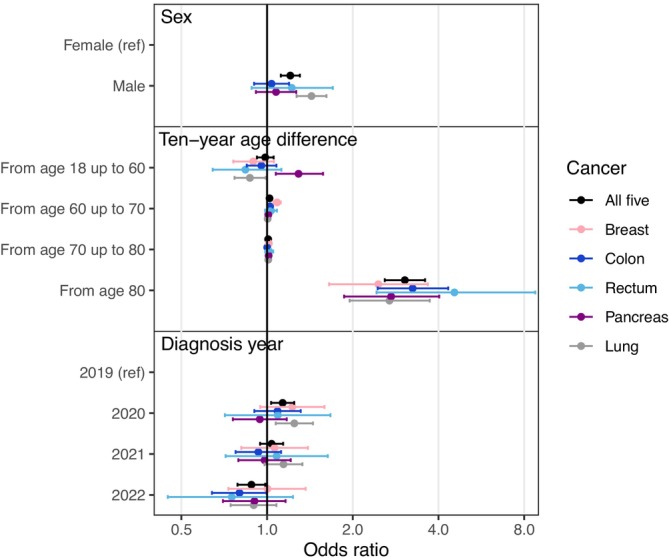
Odds ratios (and accompanying 95% confidence intervals denoted as error bars) for emergency presentation by demographic predictors, from multivariable models for all five sites combined and for each site individually. Odds ratios above 1 indicate an increased proportion of patients diagnosed by emergency presentation. Note the use of linear splines for age at diagnosis, with reported odds ratios for age differences relating to a 10‐year increase in age within that age category. Thus, age differences were typically small for younger patients, with the proportion diagnosed by emergency presentation increasing rapidly from age 80.

Multivariable analysis showed a clear non‐linear age association between age and proportion of patients diagnosed through emergency presentation. Overall, for patients aged 80 or younger, there appeared to be minimal difference in the risk of emergency presentation by age (Figure 1; Table S8). Above age 80, increasing age was strongly associated with increased odds of emergency presentation, with the odds ratio associated with a 10‐year increase in age of 3.1 (95% CI 2.6–3.6). For lung cancer, but not other individual sites, there was evidence that men were more likely to have been diagnosed after emergency presentation (OR 1.43, 95% CI 1.27–1.62), and there was also evidence of a higher prevalence of emergency diagnoses in 2020 (OR vs. 2019 1.24, 95% CI 1.07–1.44).

**FIGURE 2:**
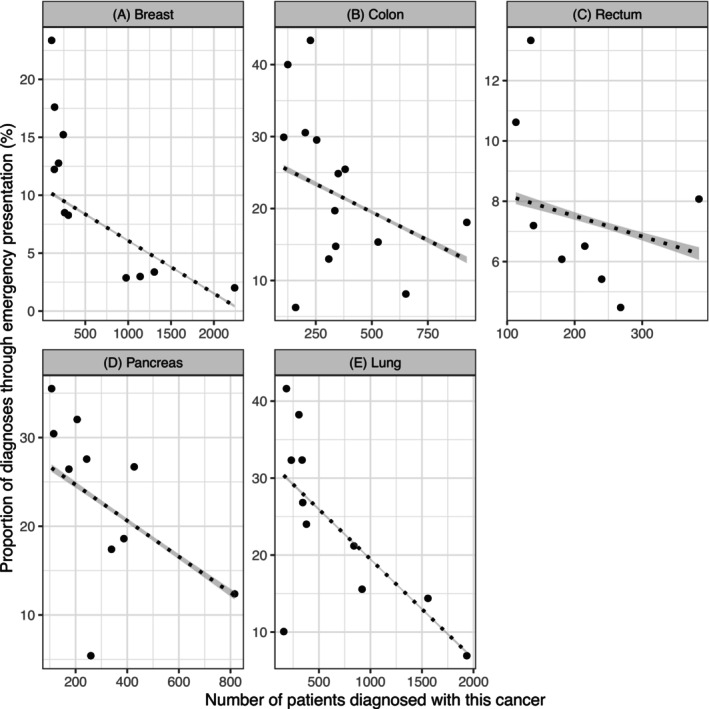
Scatter plot of percentage of patients diagnosed after an emergency presentation against number of patients with a diagnosis at the hospital for each individual cancer site, restricting only to hospitals with at least 50 diagnosed patients. Separate panels for each cancer: (A) breast; (B) colon; (C) rectum; (D) pancreas; (E) lung. Typically, there was an apparent negative correlation between the cancer‐specific number of patients diagnosed at a specific hospital and the proportion of patients diagnosed after emergency presentation. We believe this reflects a selection process; ‘routine’ diagnoses are concentrated at hospitals that treat many patients with the relevant type of cancer, while emergency diagnoses are spread relatively evenly across all hospitals.

Hospitals with a higher volume of diagnosed patients typically had lower emergency presentation rates (Figure 2). Even between hospitals with similar case volumes, there was considerable variation in emergency presentations.

### 3.1 Associations between diagnosis after emergency presentation and cancer outcomes

**FIGURE 3:**
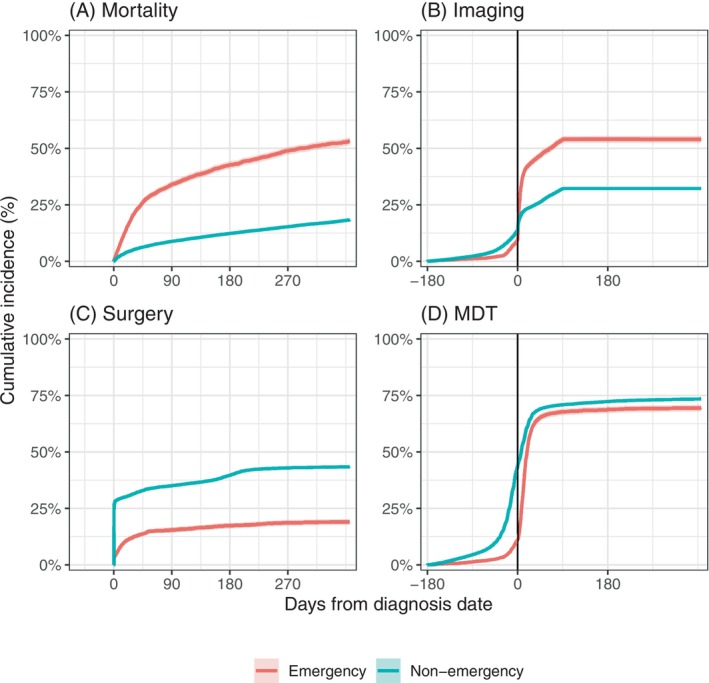
Cumulative incidence of (A) mortality (B) use of in‐hospital imaging (C) surgery (D) multi‐disciplinary team meeting discussion by emergency presentation status. For B–D, mortality is treated as a competing risk. Imaging reports are only considered up to 90 days after diagnosis. Emergency presenters were expected to have much higher mortality rates, as observed in panel A. The higher use of in‐hospital imaging post‐diagnosis (and lower use before diagnosis, panel B) is consistent with a lack of suspicion of cancer prior to the emergency presentation. The lower use of surgery (panel C) is consistent with worse health and disease among emergency presenters. The lower proportion of patients discussed at MDT before diagnosis (panel D) is again consistent with a lack of suspicion of cancer prior to emergency presentation, while the rapid equalisation in proportion discussed at MDT suggests the emergency presentation was typically part of the diagnostic process. The slight difference in the proportion of patients discussed at MDT may partly be explained by patients dying before they could be discussed.

**FIGURE 4:**
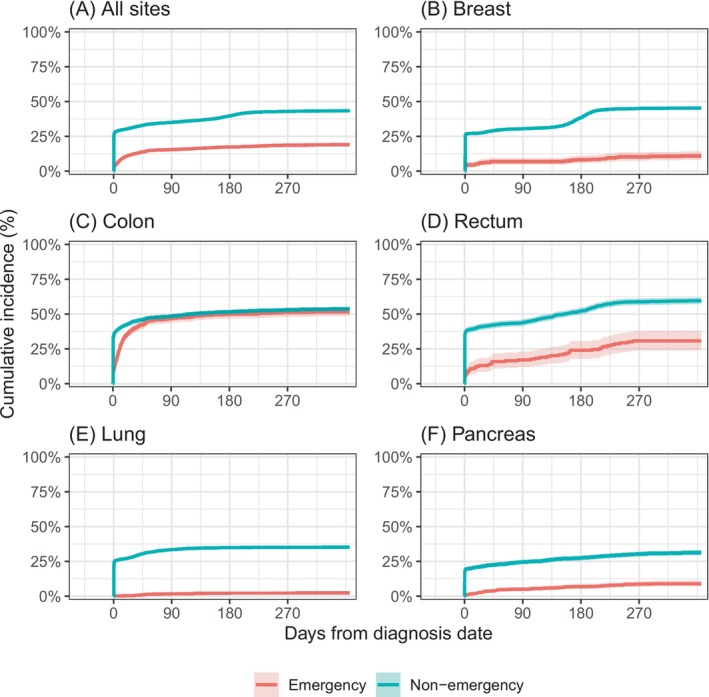
Cumulative incidence of surgery by emergency presentation status overall and for each individual cancer site. Separate panels for each cancer: (A) all five sites; (B) breast; (C) colon; (D) rectum; (E) lung; (F) pancreas. Typically, the proportion of patients receiving surgery was much lower for emergency presenters than for patients diagnosed by other routes, with differences of around 20 percentage points or more. This did not hold for colon cancer, where around half of patients received surgery regardless of the route to diagnosis.

Descriptive analysis showed far worse survival in patients diagnosed after emergency presentation than those diagnosed through other routes, with 49% vs. 16% dying within a year of diagnosis, respectively (Table 1; Figures 3A and S1). Compared to patients diagnosed through other routes, for patients diagnosed through emergency presentation, recorded imaging rates were higher after diagnosis (Figures 3B and S2), but lower before diagnosis. Surgery rates, overall across the five sites, were lower in emergency presenters compared with patients diagnosed through other routes (18% vs. 41%, respectively), and with far fewer surgeries on the day of diagnosis (Figure 3C), though with variability between cancer sites (see below, Figure 4). Finally, the proportion of patients who had been discussed at MDT prior to diagnosis was considerably lower among emergency presenters (Figures 3D and S3).

Notably, differences in surgery were specific to individual cancer sites. For colon cancer, there was no apparent difference in the use of surgery between emergency presenters and those diagnosed by other routes (Figure 4C). But for rectal cancer, and for breast, lung and pancreatic cancer, emergency presenters were far less likely to receive surgery (Figure 4). Cancer‐specific cumulative incidence curves for mortality, MDT discussion, and in‐hospital imaging are shown in Figures S1–S3.

**FIGURE 5:**
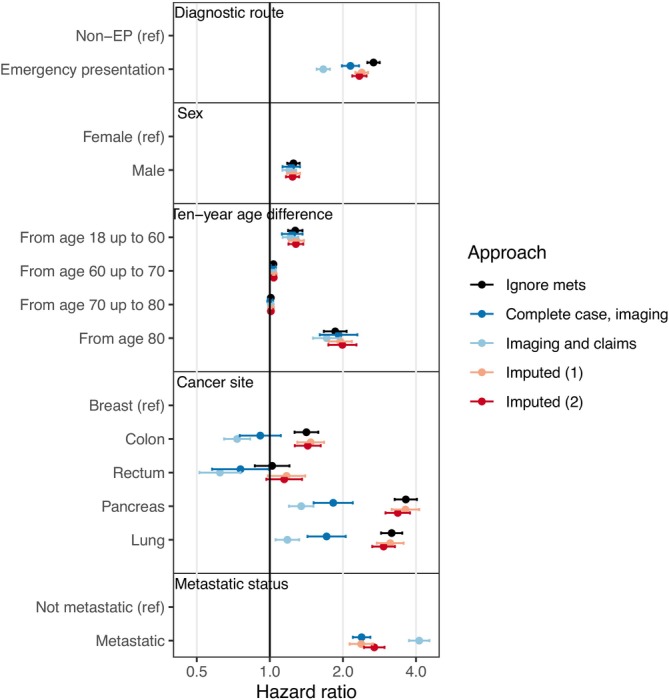
Hazard ratios from Cox proportional‐hazards regression. Hazard ratios above 1 indicate increased risk of death in the first year following diagnosis. Different coloured circles show results from models with different methods of accounting for metastatic status. Emergency presenters had worse outcomes than those diagnosed by other routes regardless of the method used to account for metastatic status.

Cox proportional‐hazards regression confirmed that worse mortality outcomes in patients diagnosed after an emergency presentation were not wholly explained by differences in cancer site or age case‐mix (Figure 5; Table S9). While older patients and those with lung and pancreatic cancer were more likely to be diagnosed following an emergency presentation and more likely to die within a year of diagnosis regardless of their diagnostic route, considerable excess hazard associated with diagnosis as an emergency remained even after adjusting for age and cancer site (HR 2.67, 95% CI 2.52–2.84). Metastatic status from imaging reports was highly incomplete in our data (34.3% completeness; Table 1). Adjusting for metastatic status reduced the association between emergency diagnosis and mortality, but the weakest adjusted association from the four different methods that included adjustment for metastatic status was 1.66 (95% CI 1.56–1.76).

## 4 DISCUSSION

One in six patients diagnosed with breast, colon, rectum, pancreas or lung cancer not previously known to AP‐HP initially presented as emergencies. These patients are less likely to be treated by surgery and far more likely to die in the first year after diagnosis than those diagnosed through other routes, even after adjustment for metastatic status.

Differences between emergency and non‐emergency presenters regarding use of in‐hospital imaging, multi‐disciplinary team meeting discussion, and receipt of surgery were all consistent with emergency presenters having little or no cancer‐directed secondary care diagnostic care activity preceding the emergency hospital admission leading to their diagnosis. For example, imaging rates were higher after diagnosis (and lower prior to diagnosis) for patients identified as emergency presenters, consistent with the hypothesis that the emergency care episode typically related to the first clinical suspicion of cancer. Emergency presenters were also less likely to have been discussed in an MDT prior to the first cancer code, again suggesting non‐elective cancer management. For surgery, we observed minimal difference for colon cancer, where emergency presentation is typically prompted by intestinal obstruction or perforation, which are life‐threatening complications requiring emergency surgical treatment that can also include tumour resection. In prior research, the definition of emergency presentation of colorectal cancer has often included the need for emergency surgery.

We analysed data from before and during the COVID‐19 pandemic, and overall found little difference in the proportion of patients diagnosed through emergency presentation during the pandemic. The key exception was lung cancer, where emergency presentations were more common in 2020. Given certain common respiratory symptoms between lung cancer and COVID‐19 and the fact that imaging was used as a triage procedure for the latter, incidental diagnoses during COVID‐19 screening may have contributed to this finding. A separate study on the AP‐HP database we used found 72 cancer diagnoses made through COVID‐19‐related chest CT scans during the French lockdowns.^22^ The disruptions to care during the pandemic are also likely to have cancelled or delayed appointments for patients that were symptomatic, leading to an emergency presentation. A separate detailed analysis of pathways and outcomes pre‐ vs. post‐COVID outbreak for lung cancer patients,^12^ using the same dataset we analysed here, found no major difference between the two periods.

### 4.1 Strengths and limitations

While the quality of French claims data for cancer is acceptable,^23^ the use of hospital‐based (as opposed to population‐based) data introduces certain limitations. In particular, we only have diagnosis information for patients who are admitted to an AP‐HP hospital. We do not have information on diagnostic and treatment operations performed in the community, at a private hospital, or at a public hospital that is not part of AP‐HP. Therefore, our definition of ‘emergency presentation’ probably includes patients whose cancer had already been diagnosed in primary care and transferred to AP‐HP hospitals for specific care. Incorporating information from population‐based cancer registries would address these issues, but France only maintains regional registries, none of which covers the Paris region.

Because the underlying dataset is limited only to AP‐HP hospitals, there may be specific differences that would mean results are not generalisable to France as a whole. Despite this, comparison with Globocan estimates of cancer incidence by age for France suggests that AP‐HP diagnosis counts are relatively similar (Table S10).^24^ The main differences are an over‐representation of pancreatic compared to the other four cancer sites and a younger population of cancer patients than expected from incidence statistics. The difference in pancreatic cancer incidence is likely due to one hospital in AP‐HP being a major referral centre, with treatment for pancreatic cancer being particularly centralised. The age difference may reflect bias introduced by using hospital diagnoses only (with older patients referred to long‐term care facilities or hospice care) and by these university hospitals running clinical trials that may preferentially recruit younger patients, but may partly reflect the population of the Paris region being on average younger than the population of France.^25^ AP‐HP also provides many second opinions for cancer patients, typically more frequently for younger patients. Overall, we believe our results will generalise to the wider French healthcare system but highlight the need for future research using more comprehensive datasets.

The proportion of newly referred patients diagnosed after an emergency presentation was typically lower in hospitals with a high volume of patients with that cancer. This may be a real difference, as non‐emergency diagnoses of cancer would be more likely at large hospitals with specialist units because of elective referrals from other hospitals or health centres inflating the denominator of cases. In contrast, a patient diagnosed at a hospital that rarely treats their type of cancer is likely to have been diagnosed by an unusual route; a primary care physician who suspects cancer is likely to refer to a hospital that specialises in treating this cancer.

We used natural language processing to identify dates of imaging and MDT reports, and to extract information on metastatic status from these reports. This method of extracting metastatic status has been validated for the cancer sites included in this study,^11^, ^12^, ^13^, ^14^ and for lung cancer, for example, had a sensitivity of 78% and a positive predictive value of 86%,^12^ with similar performance for the other sites.

### 4.2 Comparisons with the literature

The main sources of data on the proportion of cancer patients diagnosed after an emergency presentation come from an international study describing population‐based rates of emergency presentation in six high‐income countries,^1^ and various single‐country studies in the United Kingdom,^2^, ^26^, ^27^, ^28^, ^29^ Canada,^30^ Norway,^31^, ^32^ Denmark,^33^, ^34^ Spain,^35^, ^36^ New Zealand,^37^ and the United States.^38^, ^39^

Many of our results are in line with this pre‐existing evidence. We see higher prevalence of emergency presentation in older patients and in those with cancers such as lung and pancreas which have poor prognosis and present diagnostic difficulties due to often non‐specific presenting symptoms that increase ‘diagnostic difficulty’. These findings contrast with the low prevalence of emergency presentations in breast cancer, which has a simple symptom signature typically characterised by breast lump and other organ‐specific (breast) symptoms. Additionally, we see a contrasting difference in the percentage of emergency presentations for colon and rectal cancer, an observation that is highly consistent with findings reported in several other country populations, again reflecting the substantial differences in the specificity of the typical symptoms of colon and rectal cancer.^1^, ^40^ Concordant with prior evidence, we also see that patients with metastatic cancer are more likely to have been an emergency presentation^1^, ^29^; and that emergency presentation is consistently associated with worse cancer outcomes, even after adjustment for metastatic status at diagnosis.^1^, ^27^

The main difference with prior evidence is that previous studies have typically given estimates of emergency presentation that are at least 5–10 absolute percentage points higher than we observe in our study (particularly for pancreatic, lung and colon cancer), and have reported lower prevalence of EP in men than in women for most cancer sites.^1^, ^29^ The difference in the overall rate may be explained by artefactual inflation of the denominator (total number of patients) by cases previously diagnosed elsewhere, referred to AP‐HP hospitals for trial participation or specialist management. We believe such referrals may also partly explain why age‐related associations were minimal in this study until age 80, while in prior studies in other settings, the proportion diagnosed via EP increased from around age 65.

Considering the generalisability of our findings to the broader population of patients with the five studied cancers in France, we note that our study population is relatively younger compared to the rest of French incident cases of cancer; standardising to cancers diagnosed in France increases the proportion who would be emergency presenters for each site by several percentage points (Table S11). Furthermore, it might also be that diagnostic work‐up for cancer is more readily accessible in the Paris region, a densely populated area with numerous hospitals, while we know that in other French regions travel time to the nearest cancer care centre is associated with poorer outcomes in at least some of the cancers we study (e.g., lung^41^).

Our analyses account for the potential effect of the COVID‐19 pandemic but do not explore it in detail; however, this is reported in other publications using the same data source, where pathways and outcomes are analysed pre‐ vs. post‐outbreak.^11^, ^12^, ^13^, ^14^ While we have examined associations with age, sex, cancer site, and metastatic status, international evidence suggests that socio‐economic status, in particular, is associated with emergency presentation.^27^, ^29^, ^37^, ^42^ Within France, we would expect to find that patients with higher precariousness are at higher risk of EP—but in line with the international evidence, we would still expect EP to affect all social strata. Further, it is open to debate whether the route to diagnosis is the principal ‘cause’ of the worse outcomes in emergency presenters per se, or whether worse prognosis is due to differences in socio‐economic status and the general health status of patients, or due to tumour factors. Nonetheless, some existing research explores adjusting for comorbidity when examining the prognosis of emergency presenters and suggests there is little impact.^27^

We considered several approaches to accounting for metastatic status. In‐hospital imaging was disproportionately carried out for emergency presenters in our study, while metastatic status is often underreported in claims data.^43^, ^44^ Associations obtained from imputation of metastatic status based on those with in‐hospital imaging results were similar to the complete case analysis, while associations based on both (recorded) in‐hospital imaging results and metastatic status recorded in claims data were attenuated substantially. Considering previous data on the impact of stage adjustment on survival outcomes,^1^ where adjustment reduced the hazard ratio associated with emergency presentation by around 20% to 35%, we believe the dramatic reduction in the strength of the association seen when claims data were considered in the imputation to be unrealistically large, noting, however, that strong associations (hazard ratios >2 across the 5 cancer sites, Figure 5) prevail.

The difference in results using different approaches to capturing metastatic status highlight the limitations of relying on claims data to identify metastatic status. In particular, there is a risk of ‘false positives’, particularly if patients' have non‐metastatic disease at diagnosis but progress after first‐line treatment. Typically, such patients will have very poor prognosis, leading to the apparent overadjustment which we believe we observed in this study. Relying on imaging reports is likely to be more accurate, but introduces considerable amounts of missing data due to the challenges of accessing imaging reports in non‐hospital settings. Near‐complete ascertainment of stage in large research cohorts is difficult to achieve without nationwide cancer registration systems, but where metastatic status or stage is of primary interest there may be value in producing ‘gold standard’ subcohorts for sensitivity analyses and for defining imputation models, whether these be based on cancer registry data or on manual review of the electronic health record.

Men with lung cancer at AP‐HP were considerably more likely to have been diagnosed by emergency presentation than women with lung cancer. Although such differences have been reported previously, the scale of this sex difference (+4.6 percentage points higher in men) appeared larger than for international peers,^1^ where differences ranged from 0.7 percent in England to +3.8 percentage points in British Columbia.

### 4.3 Implications

In line with existing evidence, we show emergency presentation is a marker of poor prognosis above and beyond age or metastatic status. There are many reasons why patients may be diagnosed after an emergency presentation, and many of these are unavoidable; for example, incidental identification of cancer following an unrelated myocardial infarction or a cancer that is simply exceptionally aggressive with little or no prodromal symptoms preceding the emergency presentation. Others may be avoidable but may represent prompt diagnosis; for example, if primary care physicians refer patients to the emergency department because of delays in elective pathways for diagnostic and treatment. In other countries, socio‐economic deprivation is strongly associated with emergency presentation.^2^, ^45^ A possible hypothesis is due to socio‐economic group differences in promptness of help‐seeking once symptoms have developed, due to a range of cognitive, emotional, or practical barriers. In France, research has shown that social deprivation is an independent risk factor for morbidity after colorectal cancer surgery.^46^ More generally, social deprivation is regularly associated with poorer outcomes and care in France,^47^ and future research should aim to describe how this is associated with EP, to help us analyse the role of EP per se. In particular, the extent to which EP itself is an independent cause of worse outcomes and the extent to which observed associations are due to confounding by social deprivation or tumour factors. International evidence showing differences in rates of emergency presentation between countries and evidence from the United Kingdom showing a decrease in emergency presentation between 2006 and 2015,^29^ suggests that at least some emergency hospitalisations could be avoided as they relate to diagnostic delays introduced by patient or health system factors. Patients diagnosed after emergency presentation consistently seem to have worse outcomes, suggesting reducing emergency diagnoses (e.g., through increasing screening participation, campaigns targeted at socio‐economically deprived groups or better coordination with out‐of‐hospital care) would help increase cancer survival.

We posit that rates of emergency presentation are a useful measure of the performance of diagnostic pathways for cancer in a health system. Within AP‐HP, there is scope for research to examine the extent to which emergency presentations could have been avoided. More broadly, analysing population‐based nationwide databases to establish the frequency of emergency presentation in France, and how it varies regionally and between different patient groups, may help address inequalities in cancer care.^48^, ^49^ In the absence of a nationwide cancer registry in France, this study shows that simple claims‐based indicators are promising for routinely measuring emergency diagnosis. Future studies using French regional population‐based registries or the national claims database could help assess the generalizability of our findings.

## AUTHOR CONTRIBUTIONS

**Matthew E. Barclay:** Conceptualization; investigation; funding acquisition; writing – original draft; methodology; visualization; writing – review and editing; software; formal analysis. **Ariel Cohen:** Software; validation; data curation; resources; writing – review and editing. **Sonia Priou:** Validation; software; data curation; resources; writing – review and editing. **Marie Verdoux:** Resources; writing – review and editing. **Rémi Flicoteaux:** Resources; writing – review and editing. **Alexis Laurent:** Resources; writing – review and editing. **Gilles Chatellier:** Resources; writing – review and editing. **Christophe Tournigand:** Resources; writing – review and editing. **Georgios Lyratzopoulos:** Methodology; writing – review and editing; writing – original draft. **Emmanuelle Kempf:** Writing – original draft; writing – review and editing; resources; supervision. **Guillaume Lamé:** Conceptualization; methodology; writing – review and editing; writing – original draft; supervision.

## CONFLICT OF INTEREST STATEMENT

All authors have completed the ICMJE uniform disclosure form at http://www.icmje.org/disclosure-of-interest/ and declare: no support from any organisation for the submitted work; Matthew E. Barclay has received personal fees from Grail Inc. for membership of an Independent Data Monitoring Committee; no other relationships or activities that could appear to have influenced the submitted work.

## ETHICS STATEMENT

The study was approved by the AP‐HP's Scientific and Ethics Committee (IRB00011591; approval CSE 20‐0055_COVONCO‐AP) on May 15, 2020. The creation of AP‐HP's CDW has been authorised by the French ‘Commission Nationale Informatique et Libertés’ on 19 January 2017 (authorisation n°1980120). No data analysis for this study was performed prior to approval.

## Supporting information


**Data S1.** Supporting Information.

## Data Availability

The data that support the findings of this study are available from the AP‐HP Health Data Warehouse, and can be obtained on request from https://eds.aphp.fr/. Further information is available from the corresponding author upon request.
