# Supplementary material for: Emergency cancer diagnosis in Paris: A cross‐sectional study using AP‐HP data
Source: Int J Cancer. 2025 Aug 8;157(11):2283–93. doi: 10.1002/ijc.70056 (PMC12496007; doi:10.1002/ijc.70056)

Emergency Cancer Diagnosis in Paris: A Cross-Sectional Study Using AP-HP Data

Matthew E Barclay, Ariel Cohen, Sonia Priou, Marie Verdoux, Rémi Flicoteaux, Alexis Laurent, Gilles Chatellier, Christophe Tournigand, Georgios Lyratzopoulos, Emmanuelle Kempf, Guillaume Lame

Table of Contents

[Table S1. Cancer site codelists. 2](#_Toc204171089)

[Table S2. Codelists for surgical procedures for each cancer site. 3](#_Toc204171090)

[Table S3. Descriptive statistics for breast cancer patients. 7](#_Toc204171091)

[Table S4. Descriptive statistics for colon cancer patients. 8](#_Toc204171092)

[Table S5. Descriptive statistics for rectal cancer patients. 9](#_Toc204171093)

[Table S6. Descriptive statistics for lung cancer patients. 10](#_Toc204171094)

[Table S7. Descriptive statistics for pancreatic cancer patients. 11](#_Toc204171095)

[Table S8. Coefficients from the logistic models for odds of emergency presentation conditional on being diagnosed with cancer. 12](#_Toc204171096)

[Table S9. Coefficients from the Cox proportional-hazards regression models. 14](#_Toc204171097)

[Table S10. Comparison of distribution of cancer counts at AP-HP and Globocan estimates for France. 15](#_Toc204171098)

[Table S11. Crude, age-specific and age-standardised (to Globocan-France) estimates of rates of emergency presentation by site. 16](#_Toc204171099)

[Figure S1. Kaplan-Meier estimate of mortality (1-survival) to one year after diagnosis by emergency presentation status, overall and for each individual cancer site. Separate panels for each cancer: (A) All five sites (B) Breast; (C) Colon; (D) Rectum; (E) Lung; (F) Pancreas. 17](#_Toc204171100)

[Figure S2. Aalen-Johnansen estimate of cumulative incidence of in-hospital imaging by emergency presentation status, overall and for each individual cancer site. Separate panels for each cancer: (A) All five sites (B) Breast; (C) Colon; (D) Rectum; (E) Lung; (F) Pancreas. 18](#_Toc204171101)

[Figure S3. Aalen-Johnansen estimate of cumulative incidence of discussion at multi-disciplinary team meetings by emergency presentation status, overall and for each individual cancer site. Separate panels for each cancer: (A) All five sites (B) Breast; (C) Colon; (D) Rectum; (E) Lung; (F) Pancreas. 19](#_Toc204171102)

# Table S1. Cancer site codelists.

| Cancer site | ICD10-o2 codes |
| --- | --- |
| Breast | C50, D05 |
| Colon | C18, C19, D010, D011, D373, D374 |
| Rectum | C20, D012, D375 |
| Lung | C33, C34, D021, D022 |
| Pancreas | C25 excluding C254 |

# Table S2. Codelists for surgical procedures for each cancer site.

| **Site** | **CCAM Code** | **Description** |
| --- | --- | --- |
| Breast | QEFA001 | Tumorectomie du sein avec curage lymphonodal axillaire |
| Breast | QEFA003 | Mastectomie totale avec curages lymphonodaux axillaire et parasternal [mammaire interne] |
| Breast | QEFA004 | Tumorectomie du sein |
| Breast | QEFA005 | Mastectomie totale avec exérèse des pectoraux et curage lymphonodal axillaire |
| Breast | QEFA007 | Mastectomie souscutanée avec exérèse de la plaque aréolomamelonnaire |
| Breast | QEFA008 | Mastectomie partielle avec curage lymphonodal axillaire |
| Breast | QEFA010 | Mastectomie totale avec curages lymphonodaux axillaire et supraclaviculaire |
| Breast | QEFA012 | Mastectomie totale élargie en surface, avec autogreffe cutanée |
| Breast | QEFA013 | Mastectomie totale élargie en surface, avec lambeau pédiculé de muscle grand dorsal ou lambeau parascapulaire |
| Breast | QEFA015 | Mastectomie totale élargie en surface, avec lambeau libre musculocutané |
| Breast | QEFA017 | Mastectomie partielle |
| Breast | QEFA019 | Mastectomie totale |
| Breast | QEFA020 | Mastectomie totale avec conservation des pectoraux et curage lymphonodal axillaire |
| Lung | GFFA019 | Exérèse de lobe pulmonaire restant [Totalisation de pneumonectomie], par thoracotomie |
| Lung | GFFA026 | Lobectomie pulmonaire avec résection-anastomose de la bifurcation trachéale, par thoracotomie |
| Lung | GFFA015 | Bilobectomie pulmonaire avec résection et remplacement prothétique de la veine cave supérieure, par thoracotomie |
| Lung | GFFA034 | Bilobectomie pulmonaire avec résection d'organe et/ou de structure de voisinage, par thoracotomie |
| Lung | GFFA033 | Lobectomie pulmonaire supérieure avec résection de côte, de vertèbre, de vaisseau subclavier, exérèse de noeud [ganglion] lymphatique cervical et libération du plexus brachial, par cervicothoracotomie |
| Lung | GFFA029 | Segmentectomie pulmonaire unique ou multiple, par thoracotomie |
| Lung | GFFA013 | Lobectomie pulmonaire, par thoracotomie |
| Lung | GFFA030 | Lobectomie pulmonaire supérieure avec résection de côte et libération du plexus brachial, par cervicothoracotomie |
| Lung | GFFA031 | Bilobectomie pulmonaire avec résection-anastomose de la veine cave supérieure ou résection de l'oreillette gauche, par thoracotomie |
| Lung | GFFA010 | Bilobectomie pulmonaire avec résection de la paroi thoracique, par thoracotomie |
| Lung | GFFA009 | Lobectomie pulmonaire, par thoracotomie avec préparation par thoracoscopie |
| Lung | GFFA016 | Lobectomie pulmonaire avec résection-anastomose de la veine cave supérieure ou résection de l'oreillette gauche, par thoracotomie |
| Lung | GFFA018 | Bilobectomie pulmonaire, par thoracotomie |
| Lung | GFFA022 | Lobectomie pulmonaire avec résection d'organe et/ou de structure de voisinage, par thoracotomie |
| Lung | GFFA004 | Lobectomie pulmonaire avec résection-anastomose ou réimplantation de bronche, par thoracotomie |
| Lung | GFFA008 | Lobectomie pulmonaire supérieure avec résection de côte et libération du plexus brachial, par thoracotomie |
| Lung | GFFA023 | Bilobectomie pulmonaire avec résection-anastomose ou réimplantation de bronche, par thoracotomie |
| Lung | GFFA006 | Lobectomie pulmonaire avec résection et remplacement prothétique de la veine cave supérieure, par thoracotomie |
| Lung | GFFA027 | Lobectomie pulmonaire avec résection de la paroi thoracique, par thoracotomie |
| Lung | GFFC002 | Exérèse partielle non anatomique du poumon, par thoracoscopie |
| Lung | GFFA001 | Pneumonectomie avec exérèse totale de la plèvre [Pleuropneumonectomie], par thoracotomie |
| Lung | GFFA011 | Pneumonectomie avec résection-anastomose de la bifurcation trachéale, par thoracotomie |
| Lung | GFFA007 | Pneumonectomie avec résection d'organe et/ou de structure de voisinage, par thoracotomie |
| Lung | GFFA002 | Pneumonectomie avec résection et remplacement prothétique de la veine cave supérieure, par thoracotomie |
| Lung | GFFA012 | Pneumonectomie, par thoracotomie avec préparation par thoracoscopie |
| Lung | GFFA025 | Pneumonectomie avec résection de la paroi thoracique, par thoracotomie |
| Lung | GFFA024 | Pneumonectomie, par thoracotomie |
| Lung | GFFA028 | Pneumonectomie avec résection-anastomose de la veine cave supérieure ou résection de l'oreillette gauche, par thoracotomie |
| Lung | GFFA021 | Exérèses partielles non anatomiques multiples du poumon, par thoracotomie |
| Pancreas | HGFA014 | Exérèse de la papille duodénale majeure, par laparotomie |
| Pancreas | [HNFC001](https://www.aideaucodage.fr/ccam-hnfc001) | Exérèse de tumeur du pancréas, par coelioscopie |
| Pancreas | [HNFA005](https://www.aideaucodage.fr/ccam-hnfa005) | Exérèse de tumeur du pancréas, par laparotomie |
| Pancreas | [HNFA010](https://www.aideaucodage.fr/ccam-hnfa010) | Pancréatectomie gauche avec splénectomie [Splénopancréatectomie gauche] avec anastomose pancréatojéjunale ou pancréaticojéjunale, par laparotomie |
| Pancreas | [HNFA008](https://www.aideaucodage.fr/ccam-hnfa008) | Pancréatectomie gauche avec conservation de la rate, par laparotomie |
| Pancreas | [HNFA007](https://www.aideaucodage.fr/ccam-hnfa007) | Duodénopancréatectomie céphalique, par laparotomie |
| Pancreas | [HNFA006](https://www.aideaucodage.fr/ccam-hnfa006) | Pancréatectomie totale ou subtotale avec conservation du duodénum et splénectomie, par laparotomie |
| Pancreas | [HNFA011](https://www.aideaucodage.fr/ccam-hnfa011) | Pancréatectomie totale ou subtotale avec conservation du duodénum, sans splénectomie, par laparotomie |
| Pancreas | [HNFA004](https://www.aideaucodage.fr/ccam-hnfa004) | Duodénopancréatectomie totale avec splénectomie [Splénopancréatectomie totale], par laparotomie |
| Pancreas | [HNFC028](https://www.aideaucodage.fr/ccam-hnfc028) | Pancréatectomie gauche avec conservation de la rate, par coelioscopie |
| Pancreas | [HNFC002](https://www.aideaucodage.fr/ccam-hnfc002) | Pancréatectomie gauche avec splénectomie [Splénopancréatectomie gauche], par coelioscopie |
| Pancreas | [HNFA013](https://www.aideaucodage.fr/ccam-hnfa013) | Pancréatectomie gauche avec splénectomie [Splénopancréatectomie gauche], par laparotomie |
| Pancreas | [HNFA002](https://www.aideaucodage.fr/ccam-hnfa002) | Pancréatectomie gauche avec conservation de la rate, avec anastomose pancréatojéjunale ou pancréaticojéjunale, par laparotomie |
| Pancreas | [HNFA001](https://www.aideaucodage.fr/ccam-hnfa001) | Isthmectomie pancréatique avec rétablissement de continuité du conduit pancréatique, par laparotomie |
| Colorectal | HHFA018 | Colectomie transverse, par laparotomi |
| Colorectal | HHFA009 | Colectomie droite avec rétablissement de la continuité, par laparotomi |
| Colorectal | HHFA026 | Colectomie droite sans rétablissement de la continuité, par laparotomi |
| Colorectal | HHFA023 | Colectomie transverse, par coelioscopie ou par laparotomie avec préparation par coelioscopi |
| Colorectal | HHFA006 | Colectomie gauche avec libération de l'angle colique gauche, avec rétablissement de la continuité, par laparotomi |
| Colorectal | HHFA022 | Colectomie totale avec conservation du rectum, avec anastomose iléorectale, par laparotomi |
| Colorectal | HHFA008 | Colectomie droite avec rétablissement de la continuité, par coelioscopie ou par laparotomie avec préparation par coelioscopi |
| Colorectal | HHFA021 | Colectomie totale avec conservation du rectum, sans rétablissement de la continuité, par laparotomi |
| Colorectal | HHFA017 | Colectomie gauche sans libération de l'angle colique gauche, avec rétablissement de la continuité, par laparotomi |
| Colorectal | HHFA010 | Colectomie gauche sans libération de l'angle colique gauche, avec rétablissement de la continuité, par coelioscopie ou par laparotomie avec préparation par coelioscopi |
| Colorectal | HHFA014 | Colectomie gauche sans libération de l'angle colique gauche, sans rétablissement de la continuité, par laparotomi |
| Colorectal | HHFA005 | Colectomie totale avec conservation du rectum, sans rétablissement de la continuité, par coelioscopie ou par laparotomie avec préparation par coelioscopi |
| Colorectal | HHFA024 | Colectomie gauche avec libération de l'angle colique gauche, sans rétablissement de la continuité, par laparotomi |
| Colorectal | HHFA004 | Colectomie totale avec conservation du rectum, avec anastomose iléorectale, par coelioscopie ou par laparotomie avec préparation par coelioscopi |
| Colorectal | HHFA002 | Colectomie gauche avec libération de l'angle colique gauche, avec rétablissement de la continuité, par coelioscopie ou par laparotomie avec préparation par coelioscopi |
| Colorectal | HJFC023 | Proctectomie secondaire par coelioscopie avec anastomose iléoanale par voie transanale, après colectomie totale initiale |
| Colorectal | HJFA012 | Proctectomie secondaire par laparotomie avec anastomose iléoanale par voie transanale, après colectomie totale initiale |
| Colorectal | HHFC296 | Colectomie droite sans rétablissement de la continuité, par coelioscopie |
| Colorectal | HHFC040 | Colectomie gauche sans libération de l'angle colique gauche, sans rétablissement de la continuité, par coelioscopie |
| Colorectal | HHFA030 | Coloproctectomie totale sans rétablissement de la continuité, par laparotomi |
| Colorectal | HHFA031 | Coloproctectomie totale avec anastomose iléoanale, par laparotomi |
| Colorectal | HJFA006 | Résection rectosigmoïdienne par laparotomie, avec anastomose coloanale par voie anale ou par abord transsphinctérien |
| Colorectal | HJFA007 | Amputation du rectum, par laparotomie et par abord périnéal |
| Colorectal | HJFA019 | Amputation du rectum, par coelioscopie ou par laparotomie avec préparation par coelioscopie et par abord périnéal |
| Colorectal | HJFA005 | Amputation du rectum, par abord périnéal |
| Colorectal | HJFA003 | Exérèse de tumeur du rectum, par abord transsphinctérien |
| Colorectal | HJFA018 | Exérèse de tumeur du rectum, par abord transsacrococcygien [de Kraske] |
| Colorectal | HJFD002 | Exérèse de tumeur du rectum, par voie anale |
| Colorectal | HJFA004 | Résection rectosigmoïdienne avec anastomose colorectale infrapéritonéale, par coelioscopie ou par laparotomie avec préparation par coelioscopie |
| Colorectal | HJFA002 | Résection rectosigmoïdienne avec anastomose colorectale infrapéritonéale, par laparotomie |
| Colorectal | HJFA001 | Résection rectocolique avec abaissement colique rétrorectal par laparotomie, avec anastomose colorectale par voie anale |
| Colorectal | HJFA015 | Résection rectocolique totale pour aganglionose congénitale par laparotomie, avec rétablissement de la continuité par laparotomie ou par voie anale |
| Colorectal | HJFA016 | Résection rectocolique subtotale pour aganglionose congénitale par laparotomie, avec rétablissement de la continuité par laparotomie ou par voie anale |
| Colorectal | HHFA029 | Coloproctectomie totale sans rétablissement de la continuité, par coelioscopie ou par laparotomie avec préparation par coelioscopi |
| Colorectal | HJFA017 | Résection rectosigmoïdienne par coelioscopie ou par laparotomie avec préparation par coelioscopie, avec anastomose coloanale par voie anale |
| Colorectal | HHFA028 | Coloproctectomie totale avec anastomose iléoanale, par coelioscopie ou par laparotomie avec préparation par coelioscopi |
| Colorectal | HJFA004 | Résection rectosigmoïdienne avec anastomose colorectale infrapéritonéale, par coelioscopie ou par laparotomie avec préparation par coelioscopie |
| Colorectal | HJFA001 | Résection rectocolique avec abaissement colique rétrorectal par laparotomie, avec anastomose colorectale par voie anale |
| Colorectal | HJFA002 | Résection rectosigmoïdienne avec anastomose colorectale infrapéritonéale, par laparotomie |
| Colorectal | HJFA006 | Résection rectosigmoïdienne par laparotomie, avec anastomose coloanale par voie anale ou par abord transsphinctérien |
| Colorectal | HJFA007 | Amputation du rectum, par laparotomie et par abord périnéal |
| Colorectal | HJFA011 | Résection rectosigmoïdienne dépassant le cul-de-sac de Douglas, sans rétablissement de la continuité, par laparotomie |
| Colorectal | HJFA012 | Proctectomie secondaire par laparotomie avec anastomose iléoanale par voie transanale, après colectomie totale initiale |
| Colorectal | HJFA014 | Exérèse de moignon rectal résiduel, par abord périnéal |
| Colorectal | HJFA017 | Résection rectosigmoïdienne par coelioscopie ou par laparotomie avec préparation par coelioscopie, avec anastomose coloanale par voie anale |
| Colorectal | HJFA019 | Amputation du rectum, par coelioscopie ou par laparotomie avec préparation par coelioscopie et par abord périnéal |
| Colorectal | HJFC023 | Proctectomie secondaire par coelioscopie avec anastomose iléoanale par voie transanale, après colectomie totale initiale |
| Colorectal | HJFC031 | Résection rectosigmoïdienne dépassant le cul-de-sac de Douglas, sans rétablissement de la continuité, par coelioscopie |
| Colorectal | HJFA008 | Résection circonférentielle de la muqueuse d'un prolapsus rectal et plicature de la musculeuse, par abord périnéal |

# Table S3. Descriptive statistics for breast cancer patients.

|  |  | **All patients** | | **Non-emergency** | | **Diagnosed after emergency admission** | |
| --- | --- | --- | --- | --- | --- | --- | --- |
|  |  | **N** | **(col %)** | **N** | **(row %)** | **N** | **(row %)** |
| Total |  | 7,492 |  | 7,055 | (94.2%) | 437 | (5.8%) |
| Age |  |  |  |  |  |  |  |
|  | 18-64 | 4,429 | (59.1%) | 4,273 | (96.5%) | 156 | (3.5%) |
|  | 65-74 | 1,673 | (22.3%) | 1,581 | (94.5%) | 92 | (5.5%) |
|  | 75-84 | 947 | (12.6%) | 854 | (90.2%) | 93 | (9.8%) |
|  | 85-99 | 443 | (5.9%) | 347 | (78.3%) | 96 | (21.7%) |
| Metastatic status (imaging reports) |  |  |  |  | **(col %)** |  | **(col %)** |
|  | Not metastatic | 353 | (50.5%) | 309 | (57.8%) | 44 | (26.8%) |
|  | Metastatic | 346 | (49.5%) | 226 | (42.2%) | 120 | (73.2%) |
|  | Unknown | 6,793 |  | 6,520 |  | 273 |  |
| Surgery within one year |  |  |  |  |  |  |  |
|  | No | 4,518 | (60.3%) | 4,125 | (58.5%) | 393 | (89.9%) |
|  | Yes | 2,974 | (39.7%) | 2,930 | (41.5%) | 44 | (10.1%) |
| Discussed at multidisciplinary team meeting |  |  |  |  |  |  |  |
|  | No | 2,063 | (27.5%) | 1,860 | (26.4%) | 203 | (46.5%) |
|  | Yes | 5,429 | (72.5%) | 5,195 | (73.6%) | 234 | (53.5%) |
| Died within one year |  |  |  |  |  |  |  |
|  | No | 6,924 | (92.4%) | 6,672 | (94.6%) | 252 | (57.7%) |
|  | Yes | 568 | (7.6%) | 383 | (5.4%) | 185 | (42.3%) |

# Table S4. Descriptive statistics for colon cancer patients.

|  |  | **All patients** | | **Non-emergency** | | **Diagnosed after emergency admission** | |
| --- | --- | --- | --- | --- | --- | --- | --- |
|  |  | **N** | **(col %)** | **N** | **(row %)** | **N** | **(row %)** |
| Total |  | 5,147 |  | 4,104 | (79.7%) | 1,043 | (20.3%) |
| Sex |  |  |  |  |  |  |  |
|  | Female | 2,423 | (47.1%) | 1,910 | (78.8%) | 513 | (21.2%) |
|  | Male | 2,724 | (52.9%) | 2,194 | (80.5%) | 530 | (19.5%) |
| Age |  |  |  |  |  |  |  |
|  | 18-64 | 1,979 | (38.4%) | 1,673 | (84.5%) | 306 | (15.5%) |
|  | 65-74 | 1,464 | (28.4%) | 1,216 | (83.1%) | 248 | (16.9%) |
|  | 75-84 | 1,060 | (20.6%) | 841 | (79.3%) | 219 | (20.7%) |
|  | 85-99 | 644 | (12.5%) | 374 | (58.1%) | 270 | (41.9%) |
| Metastatic status (imaging reports) |  |  |  |  | **(col %)** |  | **(col %)** |
|  | Not metastatic | 1,102 | (53.6%) | 836 | (54.6%) | 266 | (50.6%) |
|  | Metastatic | 955 | (46.4%) | 695 | (45.4%) | 260 | (49.4%) |
|  | Unknown | 3,090 |  | 2,573 |  | 517 |  |
| Surgery within one year |  |  |  |  |  |  |  |
|  | No | 2,541 | (49.4%) | 2,019 | (49.2%) | 522 | (50.0%) |
|  | Yes | 2,606 | (50.6%) | 2,085 | (50.8%) | 521 | (50.0%) |
| Discussed at multidisciplinary team meeting |  |  |  |  |  |  |  |
|  | No | 1,637 | (31.8%) | 1,360 | (33.1%) | 277 | (26.6%) |
|  | Yes | 3,510 | (68.2%) | 2,744 | (66.9%) | 766 | (73.4%) |
| Died within one year |  |  |  |  |  |  |  |
|  | No | 4,262 | (82.8%) | 3,566 | (86.9%) | 696 | (66.7%) |
|  | Yes | 885 | (17.2%) | 538 | (13.1%) | 347 | (33.3%) |

# Table S5. Descriptive statistics for rectal cancer patients.

|  |  | **All patients** | | **Non-emergency** | | **Diagnosed after emergency admission** | |
| --- | --- | --- | --- | --- | --- | --- | --- |
|  |  | **N** | **(col %)** | **N** | **(row %)** | **N** | **(row %)** |
| Total |  | 1,999 |  | 1,817 | (90.9%) | 182 | (9.1%) |
| Sex |  |  |  |  |  |  |  |
|  | Female | 830 | (41.5%) | 754 | (90.8%) | 76 | (9.2%) |
|  | Male | 1,169 | (58.5%) | 1,063 | (90.9%) | 106 | (9.1%) |
| Age |  |  |  |  |  |  |  |
|  | 18-64 | 918 | (45.9%) | 859 | (93.6%) | 59 | (6.4%) |
|  | 65-74 | 563 | (28.2%) | 529 | (94.0%) | 34 | (6.0%) |
|  | 75-84 | 349 | (17.5%) | 309 | (88.5%) | 40 | (11.5%) |
|  | 85-99 | 169 | (8.5%) | 120 | (71.0%) | 49 | (29.0%) |
| Metastatic status (imaging reports) |  |  |  |  | **(col %)** |  | **(col %)** |
|  | Not metastatic | 452 | (66.7%) | 398 | (68.0%) | 54 | (58.1%) |
|  | Metastatic | 226 | (33.3%) | 187 | (32.0%) | 39 | (41.9%) |
|  | Unknown | 1,321 |  | 1,232 |  | 89 |  |
| Surgery within one year |  |  |  |  |  |  |  |
|  | No | 949 | (47.5%) | 818 | (45.0%) | 131 | (72.0%) |
|  | Yes | 1,050 | (52.5%) | 999 | (55.0%) | 51 | (28.0%) |
| Discussed at multidisciplinary team meeting |  |  |  |  |  |  |  |
|  | No | 634 | (31.7%) | 559 | (30.8%) | 75 | (41.2%) |
|  | Yes | 1,365 | (68.3%) | 1,258 | (69.2%) | 107 | (58.8%) |
| Died within one year |  |  |  |  |  |  |  |
|  | No | 1,796 | (89.8%) | 1,679 | (92.4%) | 117 | (64.3%) |
|  | Yes | 203 | (10.2%) | 138 | (7.6%) | 65 | (35.7%) |

# Table S6. Descriptive statistics for lung cancer patients.

|  |  | **All patients** | | **Non-emergency** | | **Diagnosed after emergency admission** | |
| --- | --- | --- | --- | --- | --- | --- | --- |
|  |  | **N** | **(col %)** | **N** | **(row %)** | **N** | **(row %)** |
| Total |  | 7,724 |  | 6,232 | (80.7%) | 1,492 | (19.3%) |
| Sex |  |  |  |  |  |  |  |
|  | Female | 3,059 | (39.6%) | 2,554 | (83.5%) | 505 | (16.5%) |
|  | Male | 4,665 | (60.4%) | 3,678 | (78.8%) | 987 | (21.2%) |
| Age |  |  |  |  |  |  |  |
|  | 18-64 | 3,045 | (39.4%) | 2,500 | (82.1%) | 545 | (17.9%) |
|  | 65-74 | 2,679 | (34.7%) | 2,220 | (82.9%) | 459 | (17.1%) |
|  | 75-84 | 1,542 | (20.0%) | 1,227 | (79.6%) | 315 | (20.4%) |
|  | 85-99 | 458 | (5.9%) | 285 | (62.2%) | 173 | (37.8%) |
| Metastatic status (imaging reports) |  |  |  |  | **(col %)** |  | **(col %)** |
|  | Not metastatic | 1,682 | (47.9%) | 1,361 | (51.9%) | 321 | (36.1%) |
|  | Metastatic | 1,827 | (52.1%) | 1,260 | (48.1%) | 567 | (63.9%) |
|  | Unknown | 4,215 |  | 3,611 |  | 604 |  |
| Surgery within one year |  |  |  |  |  |  |  |
|  | No | 5,591 | (72.4%) | 4,132 | (66.3%) | 1,459 | (97.8%) |
|  | Yes | 2,133 | (27.6%) | 2,100 | (33.7%) | 33 | (2.2%) |
| Discussed at multidisciplinary team meeting |  |  |  |  |  |  |  |
|  | No | 2,208 | (28.6%) | 1,767 | (28.4%) | 441 | (29.6%) |
|  | Yes | 5,516 | (71.4%) | 4,465 | (71.6%) | 1,051 | (70.4%) |
| Died within one year |  |  |  |  |  |  |  |
|  | No | 5,319 | (68.9%) | 4,700 | (75.4%) | 619 | (41.5%) |
|  | Yes | 2,405 | (31.1%) | 1,532 | (24.6%) | 873 | (58.5%) |

# Table S7. Descriptive statistics for pancreatic cancer patients.

|  |  | **All patients** | | **Non-emergency** | | **Diagnosed after emergency admission** | |
| --- | --- | --- | --- | --- | --- | --- | --- |
|  |  | **N** | **(col %)** | **N** | **(row %)** | **N** | **(row %)** |
| Total |  | 3,483 |  | 2,677 | (76.9%) | 806 | (23.1%) |
| Sex |  |  |  |  |  |  |  |
|  | Female | 1,673 | (48.0%) | 1,279 | (76.4%) | 394 | (23.6%) |
|  | Male | 1,810 | (52.0%) | 1,398 | (77.2%) | 412 | (22.8%) |
| Age |  |  |  |  |  |  |  |
|  | 18-64 | 1,305 | (37.5%) | 1,067 | (81.8%) | 238 | (18.2%) |
|  | 65-74 | 1,082 | (31.1%) | 865 | (79.9%) | 217 | (20.1%) |
|  | 75-84 | 794 | (22.8%) | 583 | (73.4%) | 211 | (26.6%) |
|  | 85-99 | 302 | (8.7%) | 162 | (53.6%) | 140 | (46.4%) |
| Metastatic status (imaging reports) |  |  |  |  | **(col %)** |  | **(col %)** |
|  | Not metastatic | 954 | (49.4%) | 786 | (52.4%) | 168 | (39.2%) |
|  | Metastatic | 976 | (50.6%) | 715 | (47.6%) | 261 | (60.8%) |
|  | Unknown | 1,553 |  | 1,176 |  | 377 |  |
| Surgery within one year |  |  |  |  |  |  |  |
|  | No | 2,629 | (75.5%) | 1,890 | (70.6%) | 739 | (91.7%) |
|  | Yes | 854 | (24.5%) | 787 | (29.4%) | 67 | (8.3%) |
| Discussed at multidisciplinary team meeting |  |  |  |  |  |  |  |
|  | No | 813 | (23.3%) | 570 | (21.3%) | 243 | (30.1%) |
|  | Yes | 2,670 | (76.7%) | 2,107 | (78.7%) | 563 | (69.9%) |
| Died within one year |  |  |  |  |  |  |  |
|  | No | 2,168 | (62.2%) | 1,834 | (68.5%) | 334 | (41.4%) |
|  | Yes | 1,315 | (37.8%) | 843 | (31.5%) | 472 | (58.6%) |

# Table S8. Coefficients from the logistic models for odds of emergency presentation conditional on being diagnosed with cancer.

|  |  |  | **Odds ratio** | |
| --- | --- | --- | --- | --- |
| **Cancer site** | **Term** | | **Estimate** | **(95% CI)** |
| All five | Intercept | | 0.17 | (0.15, 0.18) |
|  | Sex | |  |  |
|  |  | Female | (ref) |  |
|  |  | Male | 1.21 | (1.12, 1.30) |
|  | Ten year age difference | |  |  |
|  |  | From age 18 up to 60 | 0.98 | (0.92, 1.05) |
|  |  | From age 60 up to 70 | 1.02 | (1.01, 1.03) |
|  |  | From age 70 up to 80 | 1.01 | (1.00, 1.02) |
|  |  | From age 80 | 3.05 | (2.59, 3.59) |
|  | Diagnosis year | |  |  |
|  |  | 2019 | (ref) |  |
|  |  | 2020 | 1.13 | (1.03, 1.25) |
|  |  | 2021 | 1.04 | (0.94, 1.14) |
|  |  | 2022 | 0.88 | (0.79, 0.99) |
|  | Cancer site | |  |  |
|  |  | Breast | 0.29 | (0.25, 0.32) |
|  |  | Colon | 0.94 | (0.86, 1.03) |
|  |  | Rectum | 0.40 | (0.33, 0.46) |
|  |  | Pancreas | 1.20 | (1.09, 1.33) |
|  |  | Lung | (ref) |  |
| Breast | Intercept | | 0.03 | (0.03, 0.04) |
|  | Ten year age difference | |  |  |
|  |  | From age 18 up to 60 | 0.89 | (0.76, 1.06) |
|  |  | From age 60 up to 70 | 1.08 | (1.05, 1.12) |
|  |  | From age 70 up to 80 | 1.01 | (0.99, 1.03) |
|  |  | From age 80 | 2.46 | (1.65, 3.67) |
|  | Diagnosis year | |  |  |
|  |  | 2019 | (ref) |  |
|  |  | 2020 | 1.23 | (0.95, 1.59) |
|  |  | 2021 | 1.06 | (0.81, 1.39) |
|  |  | 2022 | 1.00 | (0.73, 1.37) |
| Lung | Intercept | | 0.15 | (0.13, 0.17) |
|  | Sex | |  |  |
|  |  | Female | (ref) |  |
|  |  | Male | 1.43 | (1.27, 1.62) |
|  | Ten year age difference | |  |  |
|  |  | From age 18 up to 60 | 0.87 | (0.77, 0.99) |
|  |  | From age 60 up to 70 | 1.00 | (0.99, 1.02) |
|  |  | From age 70 up to 80 | 1.01 | (1.00, 1.02) |
|  |  | From age 80 | 2.69 | (1.95, 3.73) |
|  | Diagnosis year | |  |  |
|  |  | 2019 | (ref) |  |
|  |  | 2020 | 1.25 | (1.07, 1.45) |
|  |  | 2021 | 1.14 | (0.98, 1.33) |
|  |  | 2022 | 0.90 | (0.74, 1.08) |
| Colon | Intercept | | 0.18 | (0.15, 0.22) |
|  | Sex | |  |  |
|  |  | Female | (ref) |  |
|  |  | Male | 1.04 | (0.90, 1.19) |
|  | Ten year age difference | |  |  |
|  |  | From age 18 up to 60 | 0.95 | (0.85, 1.08) |
|  |  | From age 60 up to 70 | 1.03 | (1.00, 1.05) |
|  |  | From age 70 up to 80 | 1.00 | (0.99, 1.01) |
|  |  | From age 80 | 3.25 | (2.45, 4.33) |
|  | Diagnosis year | |  |  |
|  |  | 2019 | (ref) |  |
|  |  | 2020 | 1.09 | (0.90, 1.31) |
|  |  | 2021 | 0.93 | (0.77, 1.12) |
|  |  | 2022 | 0.80 | (0.64, 1.00) |
| Rectum | Intercept | | 0.05 | (0.03, 0.08) |
|  | Sex | |  |  |
|  |  | Female |  |  |
|  |  | Male | 1.22 | (0.88, 1.70) |
|  | Ten year age difference | |  |  |
|  |  | From age 18 up to 60 | 0.84 | (0.64, 1.12) |
|  |  | From age 60 up to 70 | 1.03 | (0.98, 1.08) |
|  |  | From age 70 up to 80 | 1.02 | (0.99, 1.05) |
|  |  | From age 80 | 4.55 | (2.42, 8.75) |
|  | Diagnosis year | |  |  |
|  |  | 2019 | (ref) |  |
|  |  | 2020 | 1.09 | (0.71, 1.67) |
|  |  | 2021 | 1.08 | (0.71, 1.63) |
|  |  | 2022 | 0.75 | (0.45, 1.23) |
| Pancreas | Intercept | | 0.25 | (0.20, 0.31) |
|  | Sex | |  |  |
|  |  | Female | (ref) |  |
|  |  | Male | 1.07 | (0.91, 1.27) |
|  | Ten year age difference | |  |  |
|  |  | From age 18 up to 60 | 1.29 | (1.07, 1.57) |
|  |  | From age 60 up to 70 | 1.01 | (0.99, 1.03) |
|  |  | From age 70 up to 80 | 1.01 | (1.00, 1.03) |
|  |  | From age 80 | 2.73 | (1.86, 4.03) |
|  | Diagnosis year | |  |  |
|  |  | 2019 | (ref) |  |
|  |  | 2020 | 0.94 | (0.76, 1.17) |
|  |  | 2021 | 0.98 | (0.79, 1.21) |
|  |  | 2022 | 0.90 | (0.70, 1.16) |

# Table S9. Coefficients from the Cox proportional-hazards regression models.

|  |  | **Ignoring mets** | | **Complete case, imaging** | | **Imaging and claims** | | **Imputed (1)** | | **Imputed (2)** | |
| --- | --- | --- | --- | --- | --- | --- | --- | --- | --- | --- | --- |
| Term | | **HR** | **(95% CI)** | **HR** | **(95% CI)** | **HR** | **(95% CI)** | **HR** | **(95% CI)** | **HR** | **(95% CI)** |
| Diagnostic route | |  |  |  |  |  |  |  |  |  |  |
|  | Not-EP | (ref) |  | (ref) |  | (ref) |  | (ref) |  | (ref) |  |
|  | Emergency presentation | 2.67 | (2.52, 2.84) | 2.15 | (1.98, 2.33) | 1.66 | (1.56, 1.77) | 2.39 | (2.25, 2.54) | 2.34 | (2.19, 2.50) |
| Sex |  |  |  |  |  |  |  |  |  |  |  |
|  | Female | (ref) |  | (ref) |  | (ref) |  | (ref) |  | (ref) |  |
|  | Male | 1.25 | (1.18, 1.33) | 1.23 | (1.13, 1.33) | 1.20 | (1.13, 1.28) | 1.25 | (1.18, 1.33) | 1.24 | (1.17, 1.32) |
| Ten year age difference | |  |  |  |  |  |  |  |  |  |  |
|  | From age 18 up to 60 | 1.27 | (1.19, 1.36) | 1.24 | (1.12, 1.36) | 1.22 | (1.13, 1.31) | 1.29 | (1.20, 1.38) | 1.28 | (1.19, 1.37) |
|  | From age 60 up to 70 | 1.03 | (1.03, 1.04) | 1.03 | (1.02, 1.04) | 1.03 | (1.02, 1.04) | 1.04 | (1.03, 1.05) | 1.04 | (1.03, 1.05) |
|  | From age 70 up to 80 | 1.01 | (1.00, 1.01) | 1.00 | (1.00, 1.01) | 1.01 | (1.00, 1.02) | 1.01 | (1.01, 1.02) | 1.01 | (1.00, 1.02) |
|  | From age 80 | 1.86 | (1.67, 2.07) | 1.92 | (1.61, 2.29) | 1.71 | (1.51, 1.95) | 1.95 | (1.75, 2.18) | 1.99 | (1.74, 2.27) |
| Cancer site | |  |  |  |  |  |  |  |  |  |  |
|  | Breast | (ref) |  | (ref) |  | (ref) |  | (ref) |  | (ref) |  |
|  | Colon | 1.41 | (1.26, 1.58) | 0.91 | (0.75, 1.11) | 0.73 | (0.65, 0.83) | 1.47 | (1.30, 1.67) | 1.43 | (1.27, 1.62) |
|  | Rectum | 1.02 | (0.87, 1.21) | 0.76 | (0.58, 0.99) | 0.62 | (0.51, 0.76) | 1.17 | (0.98, 1.40) | 1.15 | (0.97, 1.36) |
|  | Pancreas | 3.63 | (3.27, 4.04) | 1.82 | (1.52, 2.20) | 1.35 | (1.20, 1.51) | 3.62 | (3.18, 4.12) | 3.36 | (2.99, 3.78) |
|  | Lung | 3.18 | (2.87, 3.51) | 1.71 | (1.43, 2.05) | 1.18 | (1.06, 1.32) | 3.13 | (2.76, 3.56) | 2.94 | (2.64, 3.27) |
| Metastatic status | |  |  |  |  |  |  |  |  |  |  |
|  | Not metastatic | NA | | (ref) |  | (ref) |  | (ref) |  | (ref) |  |
|  | Metastatic | NA | | 2.39 | (2.20, 2.60) | 4.12 | (3.74, 4.54) | 2.38 | (2.13, 2.65) | 2.69 | (2.44, 2.97) |

# Table S10. Comparison of distribution of cancer counts at AP-HP and Globocan estimates for France.

| Site | Age | Globocan | | | AP-HP | | |
| --- | --- | --- | --- | --- | --- | --- | --- |
|  |  | N | col % | col %, within site | N | col % | col %, within site |
| Breast | 0-64 | 32001 | 19.2% | 55% | 4429 | 17.1% | 59% |
| Breast | 65-74 | 14205 | 8.5% | 24% | 1673 | 6.5% | 22% |
| Breast | 75-84 | 7696 | 4.6% | 13% | 947 | 3.7% | 13% |
| Breast | 85+ | 4181 | 2.5% | 7% | 443 | 1.7% | 6% |
| Lung | 0-64 | 18143 | 10.9% | 38% | 3045 | 11.8% | 39% |
| Lung | 65-74 | 16517 | 9.9% | 34% | 2679 | 10.4% | 35% |
| Lung | 75-84 | 9275 | 5.6% | 19% | 1542 | 6.0% | 20% |
| Lung | 85+ | 4364 | 2.6% | 9% | 458 | 1.8% | 6% |
| Pancreas | 0-64 | 3469 | 2.1% | 24% | 1305 | 5.0% | 37% |
| Pancreas | 65-74 | 4299 | 2.6% | 30% | 1082 | 4.2% | 31% |
| Pancreas | 75-84 | 3808 | 2.3% | 26% | 794 | 3.1% | 23% |
| Pancreas | 85+ | 2885 | 1.7% | 20% | 302 | 1.2% | 9% |
| Colon | 0-64 | 7220 | 4.3% | 24% | 1979 | 7.7% | 38% |
| Colon | 65-74 | 8334 | 5.0% | 28% | 1464 | 5.7% | 28% |
| Colon | 75-84 | 7927 | 4.8% | 27% | 1060 | 4.1% | 21% |
| Colon | 85+ | 6008 | 3.6% | 20% | 644 | 2.5% | 13% |
| Rectum | 0-64 | 4906 | 2.9% | 30% | 918 | 3.6% | 46% |
| Rectum | 65-74 | 4949 | 3.0% | 30% | 563 | 2.2% | 28% |
| Rectum | 75-84 | 3942 | 2.4% | 24% | 349 | 1.4% | 17% |
| Rectum | 85+ | 2636 | 1.6% | 16% | 169 | 0.7% | 8% |

# Table S11. Crude, age-specific and age-standardised (to Globocan-France) estimates of rates of emergency presentation by site.

| Site | Age | Cancer cases | Diagnosed by EP | Percentage EP | | |
| --- | --- | --- | --- | --- | --- | --- |
|  |  |  |  | Age-specific percentage | Crude overall percentage | Percentage standardised to Globocan |
| Breast | 0-64 | 4429 | 156 | 3.5% |  |  |
| Breast | 65-74 | 1673 | 92 | 5.5% |  |  |
| Breast | 75-84 | 947 | 93 | 9.8% |  |  |
| Breast | 85+ | 443 | 96 | 21.7% | 5.8% | 6.1% |
| Lung | 0-64 | 3045 | 545 | 17.9% |  |  |
| Lung | 65-74 | 2679 | 459 | 17.1% |  |  |
| Lung | 75-84 | 1542 | 315 | 20.4% |  |  |
| Lung | 85+ | 458 | 173 | 37.8% | 19.3% | 19.9% |
| Pancreas | 0-64 | 1305 | 238 | 18.2% |  |  |
| Pancreas | 65-74 | 1082 | 217 | 20.1% |  |  |
| Pancreas | 75-84 | 794 | 211 | 26.6% |  |  |
| Pancreas | 85+ | 302 | 140 | 46.4% | 23.1% | 26.6% |
| Colon | 0-64 | 1979 | 306 | 15.5% |  |  |
| Colon | 65-74 | 1464 | 248 | 16.9% |  |  |
| Colon | 75-84 | 1060 | 219 | 20.7% |  |  |
| Colon | 85+ | 644 | 270 | 41.9% | 20.3% | 22.7% |
| Rectum | 0-64 | 918 | 59 | 6.4% |  |  |
| Rectum | 65-74 | 563 | 34 | 6.0% |  |  |
| Rectum | 75-84 | 349 | 40 | 11.5% |  |  |
| Rectum | 85+ | 169 | 49 | 29.0% | 9.1% | 11.1% |
| Total |  | 25845 | 3960 |  | 15.3% | 15.3% |

# Figure S1. Kaplan-Meier estimate of mortality (1-survival) to one year after diagnosis by emergency presentation status, overall and for each individual cancer site. Separate panels for each cancer: (A) All five sites (B) Breast; (C) Colon; (D) Rectum; (E) Lung; (F) Pancreas.


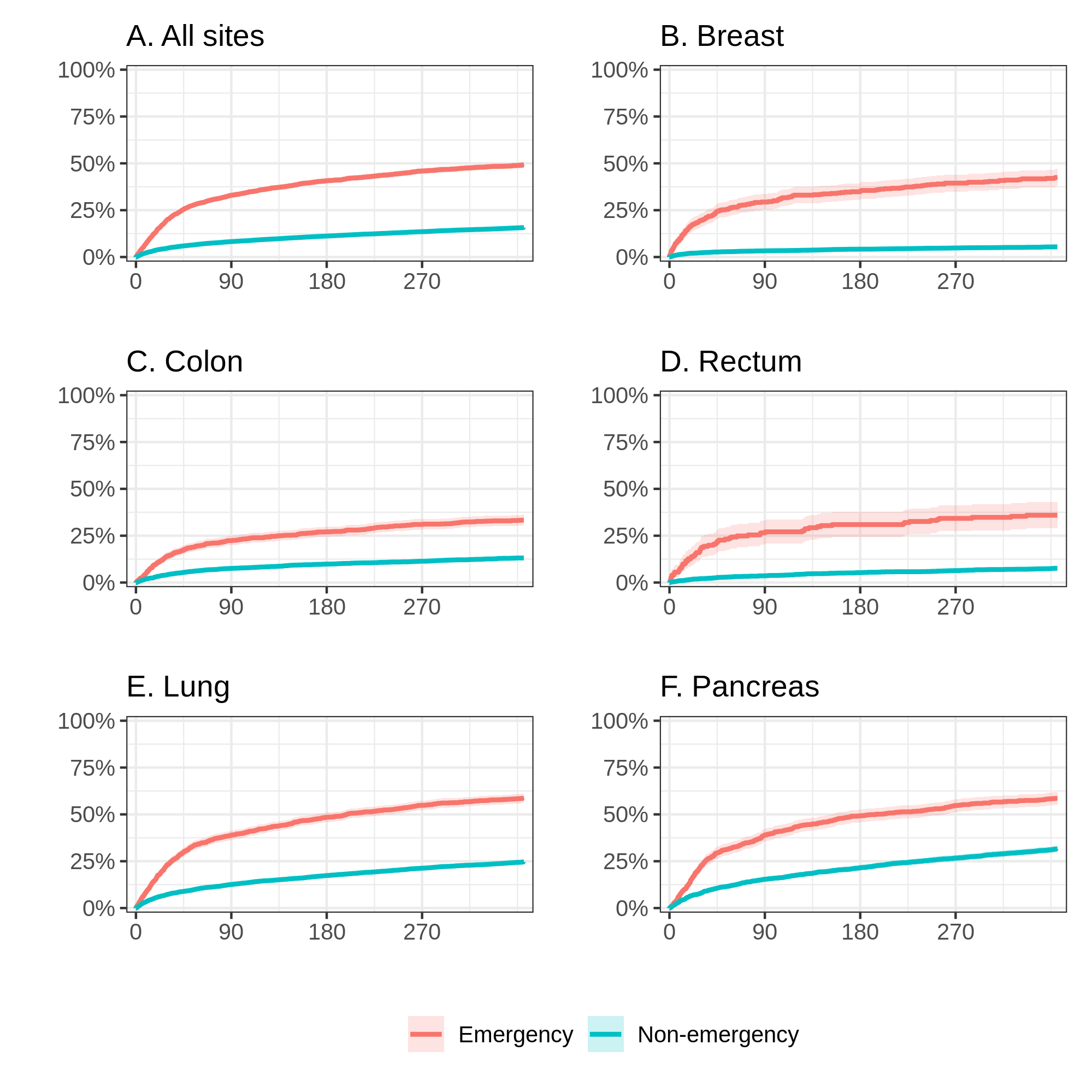


# Figure S2. Aalen-Johnansen estimate of cumulative incidence of in-hospital imaging by emergency presentation status, overall and for each individual cancer site. Separate panels for each cancer: (A) All five sites (B) Breast; (C) Colon; (D) Rectum; (E) Lung; (F) Pancreas.


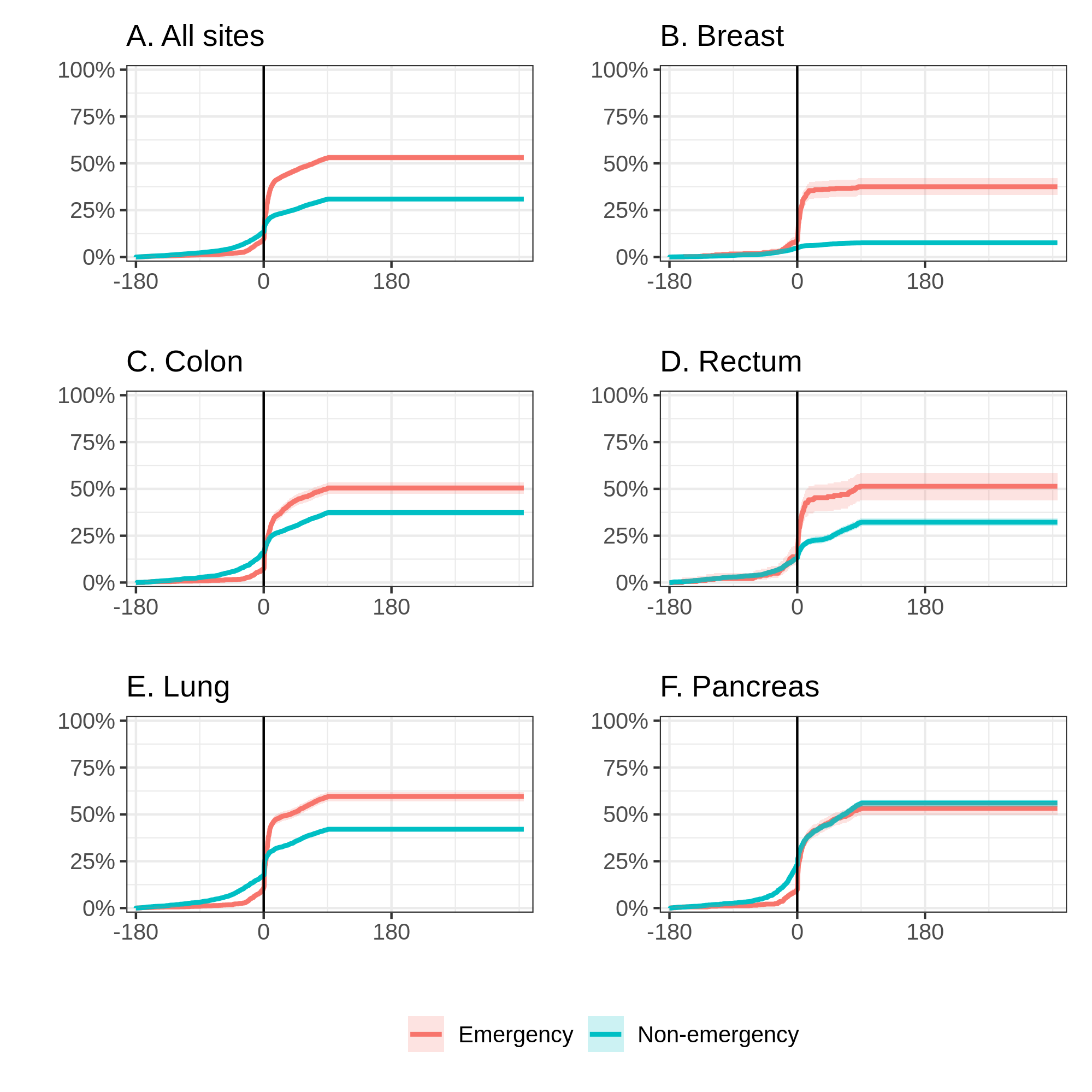


# Figure S3. Aalen-Johnansen estimate of cumulative incidence of discussion at multi-disciplinary team meetings by emergency presentation status, overall and for each individual cancer site. Separate panels for each cancer: (A) All five sites (B) Breast; (C) Colon; (D) Rectum; (E) Lung; (F) Pancreas.


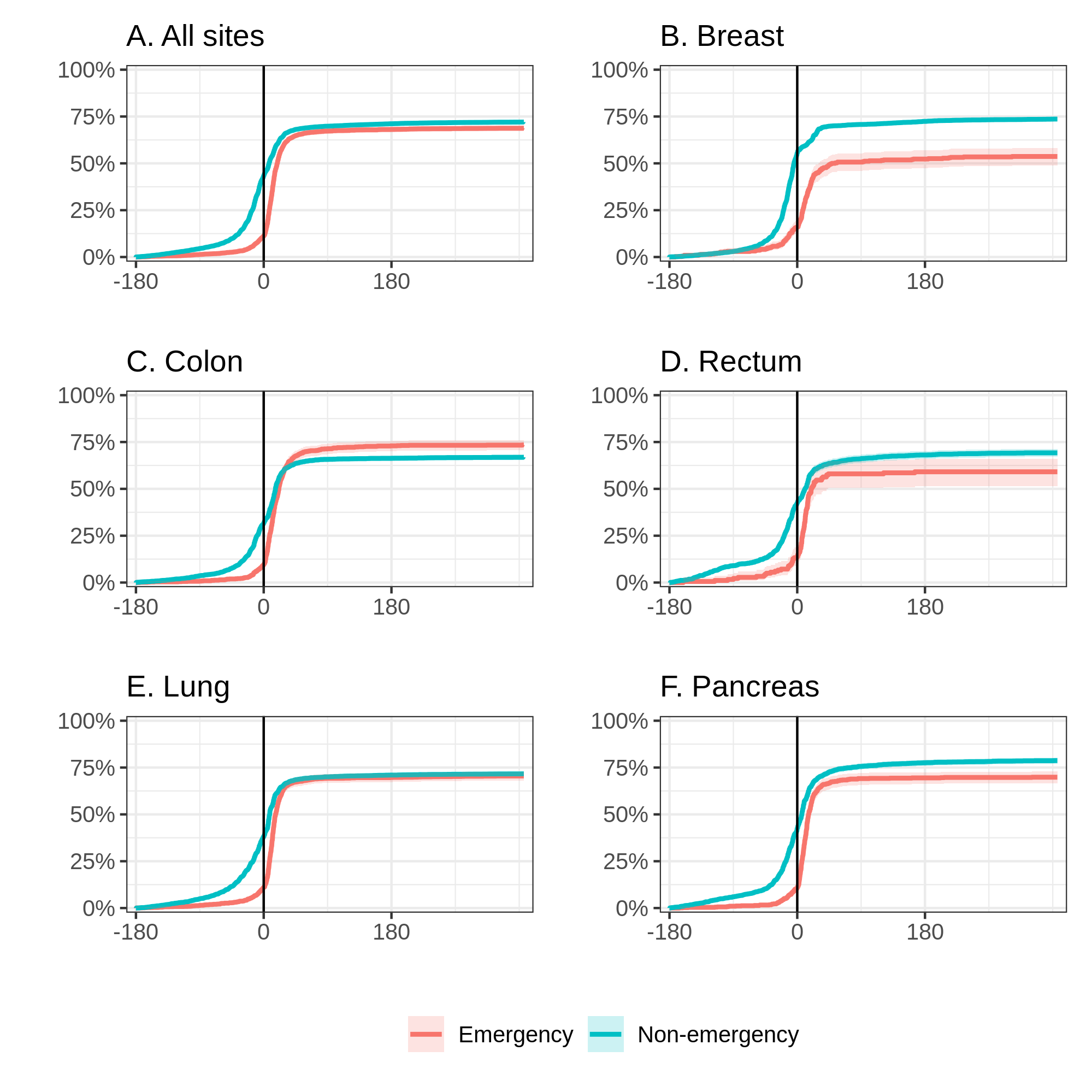

Supplement: Supplementary file 1 — Data S1. Supporting Information. [file IJC-157-2283-s001.docx]
